# Supplementary material for: Effect of Neoadjuvant Chemotherapy on Tumor-Infiltrating Lymphocytes in Resectable Gastric Cancer: Analysis from a Western Academic Center
Source: Cancers (Basel). 2024 Apr 7;16(7):1428. doi: 10.3390/cancers16071428 (PMC11010931; doi:10.3390/cancers16071428)
Supplement: Supplementary file 1 [file cancers-16-01428-s001.zip › cancers-2909200-supplementary.pdf]

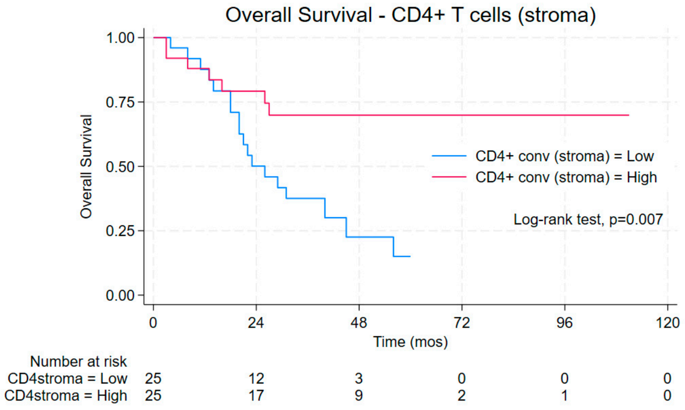

(a)

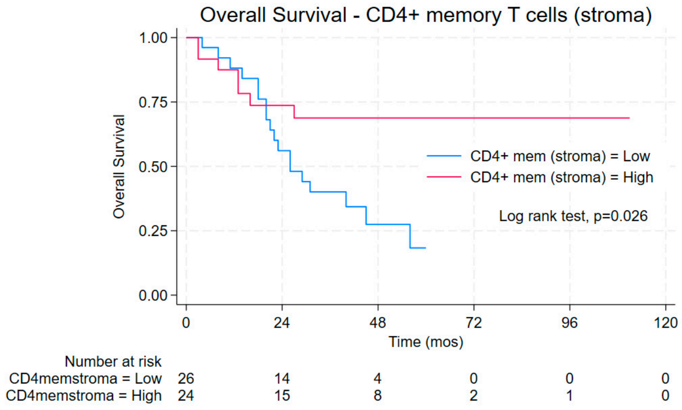

(b)

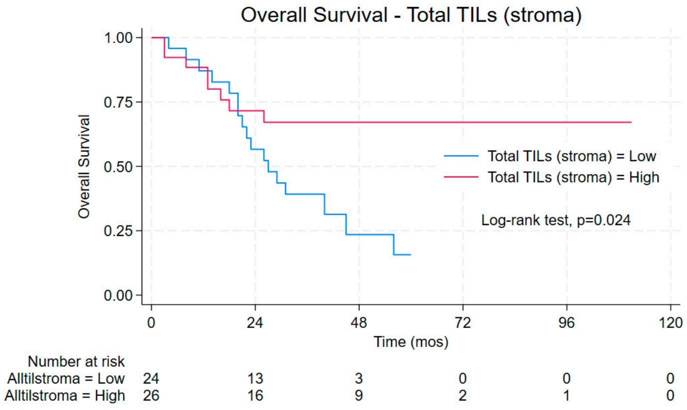

(c)

Supplemental Figure S1a-c. Kaplan-Meier survival curves with log-rank analyses demonstrating improved overall survival with high compared to low **stromal** TILs in all patients who received neoadjuvant chemotherapy. Median survival follows in parentheses. (a) low (26.0 mos) versus high (NR, not reached) CD4+ conventional (conv) T cells; p=0.007 (b) low (26.0 mos) versus high (NR) CD4+ memory (mem) T cells; p=0.026 (c) low (27.0 mos) versus high (NR) total TILs; p=0.024.

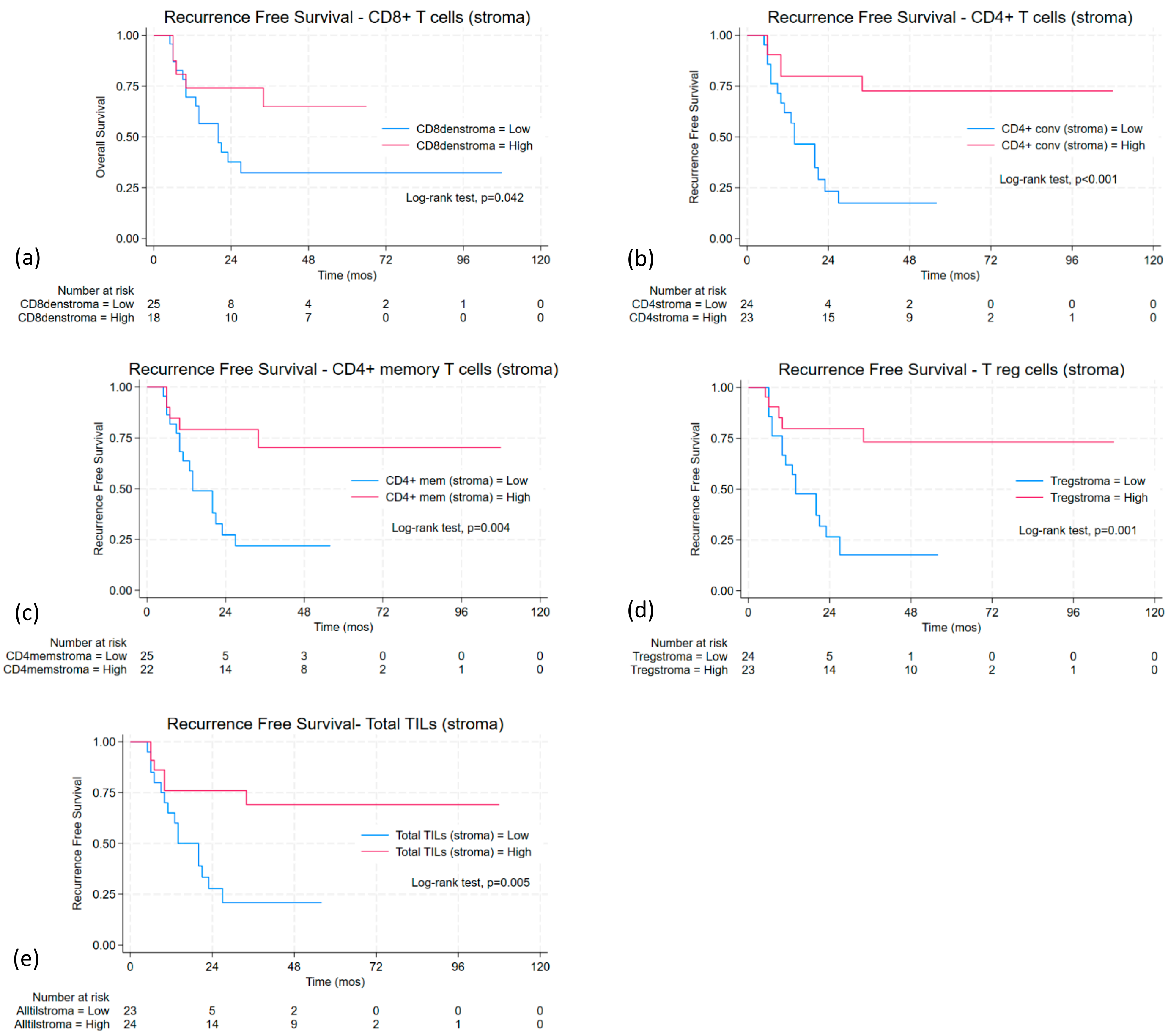

Supplemental Figure S2a-e. Kaplan-Meier survival curves with log-rank analyses demonstrating improved recurrence free survival in all patients who received neoadjuvant chemotherapy with high compared to low **stromal** TILs. Median survival follows in parentheses. (a) low (20.0 mos) versus high (NR, not reached) CD8+ conventional (conv) T cells; p=0.042 (b) low (14.0 mos) versus high (NR) CD4+ T cells; p<0.001 (c) low (14.0 mos) versus high (NR) CD4+ memory (mem) T cells; p=0.004 (d) low (14.0 mos) versus high (NR) Tregulatory (Treg) cells; p=0.001 (e) low (14.0 mos) versus high (NR) total TILs; p=0.005.

Supplemental Table S1. Multivariable Cox regression survival analysis for overall survival.

	Univariable		Multivariable	
Variable	HR (95% CI)	p	HR (95% CI)	p
ECOG				
0 (ref) v 1	1.75 (0.865-3.567)	0.119	3.079 (1.146-8.271)	**0.026**
0 v 2	0.550 (0.072-4.224)	0.566	4.248 (0.351-51.484)	0.256
Sex		0.891		
Male (ref) v Female	1.059 (0.467-2.401)			
Clinical T stage				
T1 (ref) v T2	0.703 (0.062-7.942)	0.776		
T1 v T3	1.141 (0.150-8.704)	0.899		
T1 v T4	1.524 (0.177-13.095)	0.701		
Clinical nodal stage				
N0 (ref) v N1	0.673 (0.283-1.6)	0.370		
N0 vs N2-3	0.859 (0.242-3.053)	0.815		
Clinical overall stage				
Stage I (ref) v stage II	2.661 (0.343-20.636)	0.349		
Stage I v stage III	1.948 (0.251-15.107)	0.523		
Stage I v stage IVa	0.5882 (0.360-96.173)	0.314		
NAC regimen				
FOLFOX (ref) v FLOT	1.405 (0.395-4.999)	0.600		
FOLFOX v ECF	0.920 (0.119-7.093)	0.936		
FOLFOX v EOX	1.628 (0.528-4.019)	0.396		
FOLFOX v DCF	1.159 (0.330-4.074)	0.818		
FOLFOX v ECX	0	0.986		
FOLFOX v other doublet	0.507 (0.066-3.910)	0.514		
Lymphovascular invasion				
No (ref) v Yes	2.49 (1.017-6.105)	**0.046**	2.383 (0.831-6.831)	0.106
Perineural invasion				
No (ref) v Yes	2.531 (0.964-6.646)	0.059		
Histologic subtype				
Intestinal (ref) v Diffuse	3.282 (1.113-9.681)	**0.031**	4.094 (1.128-14.854)	**0.032**
Intestinal vs mixed	7.727 (1.322-45.175)	**0.023**	36.794 (3.694-366.460)	**0.002**
Intestinal v neuorendo fx	0	0.984	0	
Tumor location				
Distal (ref) v proximal	1.786 (0.751-4.248)	0.189		
Distal v linitus plastica	2.960 (0.952-9.202)	0.061		
CD4+ conv T cells (stroma)				
Low (ref) v High	0.317 (0.131-0.766)	**0.011**	0.591 (0.216-1.615)	0.306
PD-L1 status				
Negative (ref) v positive	1.463 (0.667-3.210)	0.343		

Supplemental Table S2. Univariable and multivariable Cox regression analysis for recurrence free survival.

	Univariable		Multivariable	
Variable	HR (95% CI)	p	HR (95% CI)	p
ECOG				
0 (ref) v 1	1.927 (0.754-4.928)	0.171	2.121 (0.226-7.056)	0.214
0 v 2	3.659 (0.776-17.255)	0.101	12.763 (0.732-222.674)	0.081
Sex				
Male (ref) v Female	1.661 (0.703-3.920)	0.247		
Clinical T stage				
T1 (ref) v T2	0.821 (0.073-9.201)	0.873		
T1 v T3	1.068 (0.139-8.198)	0.949		
T1 v T4	2.080 (0.231-18.704)	0.513		
Clinical Nodal stage				
N0 (ref) v N1	1.485 (0.575-3.833)	0.414		
N0 vs N2-3	2.631 (0.678-10.206)	0.162		
Clinical overall stage				
Stage I (ref) v stage II	2.007 (0.246-16.348)	0.515		
Stage I v stage III	2.931 (0.383-22.432)	0.300		
NAC regimen				
FOLFOX (ref) v FLOT	0.966 (0.211-4.432)	0.964		
FOLFOX v ECF	2.318 (0.503-10.673)	0.281		
FOLFOX v EOX	2.718 (0.843-8.767)	0.094		
FOLFOX v DCF	0.627 (0.080-4.909)	0.657		
FOLFOX v ECX	0	0.987		
FOLFOX v other doublet	1.762 (0.383-8.110)	0.467		
Lymphovascular invasion		**0.005**		
No (ref) v Yes	4.247 (1.537-11.731)		2.989 (0.504-17.716)	0.228
Perineural invasion		**0.021**		
No (ref) v Yes	3.756 (1.225-11.519)		2.401 (0.391-14.754)	0.344
Histologic subtype				
Intestinal (ref) v Diffuse	3.994 (1.157-13.786)	**0.028**	2.444 (0.462-12.927)	0.293
Intestinal vs mixed	3.183 (0.319-31.797)	0.324	6.291 (0.329-120.136)	0.222
Intestinal v neuorendo fx	5.337 (0.543-52.419)	0.151	0.531 (0.017-16.190)	0.717
Tumor location				
Distal (ref) v proximal	1.221 (0.458-3.257)	0.690		
Distal v linitus plastica	2.682 (0.725-9.926)	0.139		
PD-L1 status				
Negative (ref) v positive	1.141 (0.480-2.714)	0.766		
CD8+ memory T cells (stroma)	0.211 (0.070-0.633)	0.006	0.408 (0.079-2.119)	0.286
CD4+ T cells (stroma)	0.206 (0.073-0.576)	0.003	0.411 (0.063-2.668)	0.351
CD4+ memory T cells (stroma)	0.258 (0.093-0.714)	0.009	0.486 (0.106-2.238)	0.355
Treg (stroma)	0.211 (0.073-0.607)	0.004	0.702 (0.173-2.842)	0.620
Total TILs (stroma)	0.277 (0.105-0.730)	0.009	1.264 (0.226-7.056)	0.790
